# Association of zinc deficiency and clinical symptoms, inflammatory markers, severity of COVID-19 in hospitalized children

**DOI:** 10.3389/fnut.2025.1566505

**Published:** 2025-03-25

**Authors:** Vita Perestiuk, Tetyana Kosovska, Liubov Volianska, Oksana Boyarchuk

**Affiliations:** Department of Children’s Diseases and Pediatric Surgery, I. Horbachevsky Ternopil National Medical University, Ternopil, Ukraine

**Keywords:** COVID-19, SARS-CoV-2 infection, zinc, children, adolescents, CRP, inflammation

## Abstract

**Introduction:**

Zinc plays an important role in the functioning of the immune system. Zinc deficiency leads to increased susceptibility to inflammatory and infectious diseases. There are few studies investigating the role of zinc in the development and progression of COVID-19 in children, and their findings remain inconsistent. This study aimed to determine the zinc levels in children with COVID-19 and assess their association with symptoms, inflammation markers, and disease progression.

**Methods:**

A prospective cohort study included hospitalized patients under 18 years who had a confirmed diagnosis of SARS-CoV-2 infection. Serum zinc concentrations were measured using a colorimetric method. Based on zinc levels, the children were divided into two groups: the first group had concentrations below 10.7 μmol/L, indicating zinc deficiency, while the second group had levels above 10.7 μmol/L, which was considered within the optimal range.

**Results:**

In total, 140 hospitalized patients with COVID-19 were examined. Zinc deficiency was identified in 40 children (28.6%), while optimal levels were found in 100 children (71.4%). Zinc status did not depend on the age of the children. Among the symptoms of acute SARS-CoV-2 infection, children with zinc deficiency showed a trend toward more frequent fever occurrences (*p* = 0.0654). No significant impact of zinc status was observed on the severity of COVID-19 or the duration of hospitalization. Children with zinc deficiency had higher median values of the neutrophil-to-lymphocyte ratio (NLR) (1.84 vs. 1.09, *p* = 0.0010), C-reactive protein (CRP) levels (9.65 vs. 3.96 mg/L, *p* = 0.0053), and fibrinogen levels (2.88 vs. 2.07 g/L, *p* = 0.0057) compared to those with adequate zinc levels. Additionally, the percentage of patients with a NLR greater than 4, elevated CRP, and fibrinogen levels was higher in the zinc-deficient group (*p* = 0.0017, *p* = 0.0107, *p* = 0.0338, respectively).

**Conclusion:**

Zinc deficiency was observed in 28.6% of children with COVID-19 and was not dependent on age. Children with hypozincemia had higher levels of inflammation markers, including the neutrophil-to-lymphocyte ratio and CRP.

## Introduction

The coronavirus disease 2019 (COVID-19) pandemic, caused by the severe acute respiratory syndrome coronavirus 2 (SARS-CoV-2), is the most significant global health crisis of the past century (1, 2). Since 2020, it has led to over 776 million reported cases and more than 7 million deaths worldwide (3).

In children, SARS-CoV-2 infection is usually mild, although more severe manifestations of the disease can sometimes develop (4, 5). The progression of COVID-19 involves complex interactions between many pathophysiological mechanisms, including the immune system’s innate and adaptive responses (6, 7). Disruption of the adaptive immune response, coupled with excessive activation of the innate immune system (inflammatory macrophages and neutrophils), contributes to severe disease outcomes (8, 9). Clinical data indicate that the severity of COVID-19 correlates with increased levels of the pro-inflammatory cytokine interleukin-6 (IL-6), elevated neutrophil-to-lymphocyte ratio (NLR), and lymphopenia. Hyperinflammation, driven by these immune changes, plays a central role in the development of acute respiratory distress syndrome and tissue damage, leading to airway collapse, multi-organ failure, and, ultimately, death in severe cases (10, 11).

Clinical or demographic risk factors for severe SARS-CoV-2 infection include older age, male sex, chronic diseases such as diabetes, cardiovascular diseases, immunosuppression, and obesity (12, 13), as well as suboptimal vitamin and micronutrient status (14–16). Key vitamins A, C, and D, and micronutrients such as selenium, copper, and zinc, which are essential for proper immune function, have been widely discussed regarding their impact on susceptibility to COVID-19 and disease progression (15, 17–20). Zinc (Zn^2+^) is an essential micronutrient necessary for supporting a variety of fundamental biological processes due to its roles as a cofactor, signaling molecule, and structural component (21, 22). Specifically, it is involved in growth development, neuro-sensory functions, deoxyribonucleic acid (DNA) synthesis, and gene expression (23). One of the most important roles of zinc in the human body is its broad impact on the immune system (21, 22), as zinc levels affect both adaptive and innate immunity. In adaptive immunity, zinc influences T-lymphocyte maturation, differentiation, and cytokine production. B-cell activation and plasma cell differentiation also depend on zinc signaling (22, 24). In innate immunity, zinc plays an anti-inflammatory role (25). Specifically, zinc deficiency is associated with higher levels of interleukin-1 (IL-1) beta and tumor necrosis factor alpha (TNF-alpha) (26), as well as altered monocyte, neutrophil, and natural killer (NK) cell activity (27). Accordingly, zinc deficiency leads to increased susceptibility to inflammatory and infectious diseases (22).

Several studies have illustrated a connection between upper respiratory tract infections, the duration of symptoms, and serum zinc levels (28, 29). The current literature on the effect of zinc on the course of COVID-19 is limited, with most of the information focusing on the adult population. For example, Vogel-González et al. (30) observed a significant association between prolonged clinical recovery, increased intensive care unit (ICU) stay, mortality, and lower zinc levels in the serum of adult patients. Chen et al. (31) conducted a retrospective review of patients with the Omicron variant of COVID-19 and found that zinc deficiency was connected with acute and persistent inflammation. However, other studies show contradictory results. Yao et al. (32) did not demonstrate a causal relationship between zinc levels and improved prognosis or survival in acute SARS-CoV-2 infection. However, studies examining the role of zinc in the development and course of COVID-19 in children are few, and their findings are inconsistent (14, 33, 34). The aim of our study was to determine the zinc levels in children with COVID-19 and assess their association with symptoms, inflammation markers, and disease progression.

## Materials and methods

### Study design

A prospective cohort study included hospitalized pediatric patients with a confirmed diagnosis of SARS-CoV-2 infection through polymerase chain reaction (PCR), rapid tests, or serological methods (detection of IgM), which were used interchangeably.

The study was conducted from September 2022 to March 2024 in the pediatric infectious diseases department of Municipal City Hospital №2 in Ternopil, Ukraine.

### Participants

The study involved hospitalized patients under 18 years who had a confirmed diagnosis of SARS-CoV-2 infection.

The inclusion criteria for the study were: age under 18 years, confirmed cases of SARS-CoV-2 infection, informed consent from parents for participation, and the ability to measure serum zinc levels during hospitalization. Exclusion criteria included the refusal of parents to consent to the study and unconfirmed cases of COVID-19.

Throughout the study, we adhered to all recommendations of the Helsinki Declaration of 1975 (as revised in 2000). The study was approved by the I. Horbachevsky Ternopil National Medical University Ethics Committee (Minutes № 70 from August 1, 2022). Upon admission, all parents or children over the age of 16 signed an informed, voluntary consent form for participation in the study, as well as for the use of diagnostic and treatment results in scientific publications.

### Data and samples collection

We carefully collected baseline and clinical data from patients upon hospital admission. Baseline characteristics included age and sex, while clinical data included comorbidities, disease severity, and duration of hospitalization. Laboratory tests upon admission included an expanded complete blood count, biochemical markers, and the determination of C-reactive protein (CRP) and ferritin levels.

COVID-19 severity was determined according to the World Health Organization (WHO) definition (35). Based on this, patients were divided into four groups: mild, moderate, severe, and critical.

Blood samples for determining serum zinc levels were collected from patients in the morning or afternoon of the same or the following day if the SARS-CoV-2 test result was positive. The samples were left to clot, then centrifuged at 4000 rpm for 10 min to separate the serum. Zinc concentration in the serum was measured using a Multiskan FC-357 microplate photometer (Thermo Fisher Scientific). A colorimetric zinc (Zn) assay kit (Elabscience E-BC-K137-M, USA) was used for the analysis. The coefficients of variation (CV) for zinc content analysis in the samples were: inter-assay CV – 0.04%, and intra-assay CV – 2.7%.

### Participants’ groups

Patients with confirmed COVID-19 were divided into two groups based on whether they had low or normal zinc levels in their serum. Currently, there is no universally accepted threshold for low serum zinc concentrations. In our study, we used 10.7 μmol/L as the cutoff for low zinc concentration in serum. Сlinical zinc deficiency was defined as a concentration of <70 μg/dL (10.7 μmol/L) (36).

### Statistical analysis

Statistical analysis of the results was performed using STATISTICA 12 software. The median and interquartile range (IQR) were used for non-normal distribution of data and categorical variables were expressed as frequency (percentage). Categorical variables were compared using the Chi-square test. A *p*-value of less than 0.05 was considered statistically significant and is highlighted in bold in the tables.

## Results

### Demographic and clinical characteristics of the study population

In total, 140 hospitalized patients were included in the study. Clinical and laboratory characteristics of the children with COVID-19 are presented in Table 1. The average age of the hospitalized patients was 3.56 ± 4.55 years, ranging from 1 month to 18 years. The male sex predominated over the female sex in the study population (56.4% vs. 43.6%).

**Table 1:** Clinical characteristics of the patients with COVID-19 and their dependence on serum zinc levels.

Characteristics	Total number, *n* = 140	Zinc deficiency (<10.7 μmol/L), *n* = 40	Optimal zinc levels (>10.7 μmol/L), *n* = 100	*P*-value
	Median (interquartile range, IQR) or *n* (%)			
Age of children, years	1.3 (0.7; 5.21)	1.05 (0.65; 3.7)	1.3 (0.73; 6.0)	0.5051
Gender				
Female	61 (43.6)	16 (40.0)	45 (45.0)	0.5899
Male	79 (56.4)	24 (60.0)	55 (55.0)	0.5899
Comorbid conditions	71 (50.7)	17 (42.5)	54 (54.0)	0.2189
Allergic diseases	40 (28.6)	10 (25.0)	30 (30.0)	0.5541
Nutritional disorders	31 (22.1)	6 (15.0)	25 (25.0)	0.1980
Overweight	11 (7.9)	0	11 (11.0)	**0.0289**
Obesity	9 (6.4)	1 (2.5)	8 (8.0)	0.2307
Undernutrition	11 (7.9)	5 (12.5)	6 (6.0)	0.1966
Cardiovascular pathologies	1 (0.7)	0	1 (1.0)	0.5256
Nervous system diseases	14 (10.0)	5 (12.5)	9 (9.0)	0.5329
Digestive system diseases	6 (4.3)	1 (2.5)	5 (5.0)	0.5094
Urinary system diseases	5 (3.6)	2 (5.0)	3 (3.0)	0.5646
COVID-19 severity				
Mild	127 (90.7)	38 (95.0)	89 (89.0)	0.2691
Moderate	5 (3.6)	1 (2.5)	4 (4.0)	0.6657
Severe	6 (4.3)	1 (2.5)	5 (5.0)	0.5094
Critical	2 (1.4)	0	2 (2.0)	0.3676
Duration of hospitalization, days	4.0 (3.0; 5.0)	3.0 (3.0; 5.5)	4.0 (3.0; 5.0)	0.4633
Leukocytes, 10^9^/L	5.92 (4.47; 8.71)	5.93 (4.3; 9.13)	5.88 (4.53; 8.29)	0.9555
Leukopenia, %	21/139 (15.1)	9/40 (22.5)	12/99 (12.1)	0.1219
Leukocytosis, %	17/139 (12.2)	5/40 (12.5)	12/99 (12.1)	0.9508
Neutrophils, 10^9^/L	2.37 (1.33; 3.68)	2.79 (1.85; 4.51)	2.19 (1.13; 3.68)	0.1055
Neutrophilia, %	8/134 (6.0)	3/39 (7.7)	5/95 (5.3)	0.5898
Lymphocytes, 10^9^/L	2.18 (1.25; 3.92)	1.88 (0.87; 3.93)	2.29 (1.42; 3.92)	0.0897
Lymphopenia	62/134 (46.3)	21/39 (53.9)	41/95 (43.2)	0.2597
Neutrophil-to-lymphocyte ratio (NLR)	1.16 (0.44; 2.67)	1.84 (0.63; 4.22)	1.09 (0.39; 2.27)	**0.0010**
NLR > 4, %	16/134 (11.9)	10/39 (25.6)	6/95 (6.3)	**0.0017**
C-reactive protein (CRP), mg/L	5.25 (1.37; 15.1)	9.65 (3.14; 28.03)	3.96 (0.62; 11.46)	**0.0053**
Elevated CRP, %	63/123 (51.1)	26/38 (68.4)	37/85 (43.5)	**0.0107**
Thrombocytes, 10^9^/L	243 (204; 307)	244.5 (210.5; 322.5)	240.0 (203.0; 297.0)	0.3931
Thrombocytopenia, %	10/138 (7.3)	5/40 (12.5)	5/98 (5.1)	0.1283
Thrombocytosis, %	9/138 (6.5)	5/40 (12.5)	4/98 (4.1)	0.0692
Prothrombin time (PT), sec	14.6 (13.3; 15.8)	14.6 (13.5; 16.2)	14.7 (13.3; 15.8)	0.6821
Prolonged PT (more than 15 s)	52/125 (41.6)	15/35 (42.9)	37/90 (41.1)	0.8589
Activated partial thromboplastin time (aPTT), sec	38.3 (34.2; 44.3)	38.2 (35.4; 46.8)	38.3 (33.4; 43.5)	0.3005
Prolonged aPTT (more than 35 s)	88/124 (71.0)	29/35 (82.9)	59/89 (66.3)	0.0674
Fibrinogen, g/L	2.26 (1.67; 3.05)	2.88 (1.94; 3.43)	2.07 (1.56; 2.84)	**0.0057**
More than 4 g/L	4/121 (3.3)	3/34 (8.8)	1/87 (1.2)	**0.0338**
Less than 2 g/L	51/121 (42.2)	9/34 (26.5)	42/87 (48.3)	**0.0290**
D-dimer, ng/mL	293.5 (100.0; 840.0)	346.0 (117; 840)	212.0 (87.5; 795.0)	0.2991
More than 250 ng/mL	31/59 (52.5)	13/19 (68.4)	18/40 (45.0)	0.0923
Ferritin	51.7 (29.3; 88.2)	48.7 (26.3; 86.5)	53.6 (32.2; 89.5)	0.7148
Hyperferritinemia, %	2/44 (4.6)	0/14 (0)	2/30 (6.7)	0.3227
Creatinine, μmol/L	36.0 (37.0; 47.0)	36.0 (28.5; 42.0)	36.0 (30.0; 49.0)	0.3576
Total protein, g/L	61.7 (56.0; 66.0)	61.5 (55.5; 66.5)	61.7 (57.0; 65.0)	0.8444
Hypoproteinemia, %	53/133 (39.9)	18/40 (45.0)	35/93 (37.6)	0.4262
Zinc level, μmol/L	12.3 (10.6; 14.6)	9.17 (7.94; 10.27)	13.27 (12.02; 16.11)	**<0.0001**

Statistically significant values are highlighted in bold.

Comorbid conditions were identified in more than half of the patients (50.7%). The most common comorbidities were allergic conditions (28.6%) and nutritional disorders (22.1%). Diseases of the nervous, digestive, urinary, and cardiovascular systems observed less frequently.

Mild COVID-19 was diagnosed in 90.7% of the studied children. Only 5 patients (3.6%) had a moderate course, 6 children (4.3%) had a severe course, and 2 children (1.4%) had a critical course. No deaths were observed in our sample. The average duration of hospitalization was 4.6 ± 3.4 days, ranging from 1 to 20 days.

### Zinc levels in patients during the acute phase of SARS-CoV-2 infection

**Figure 1:**
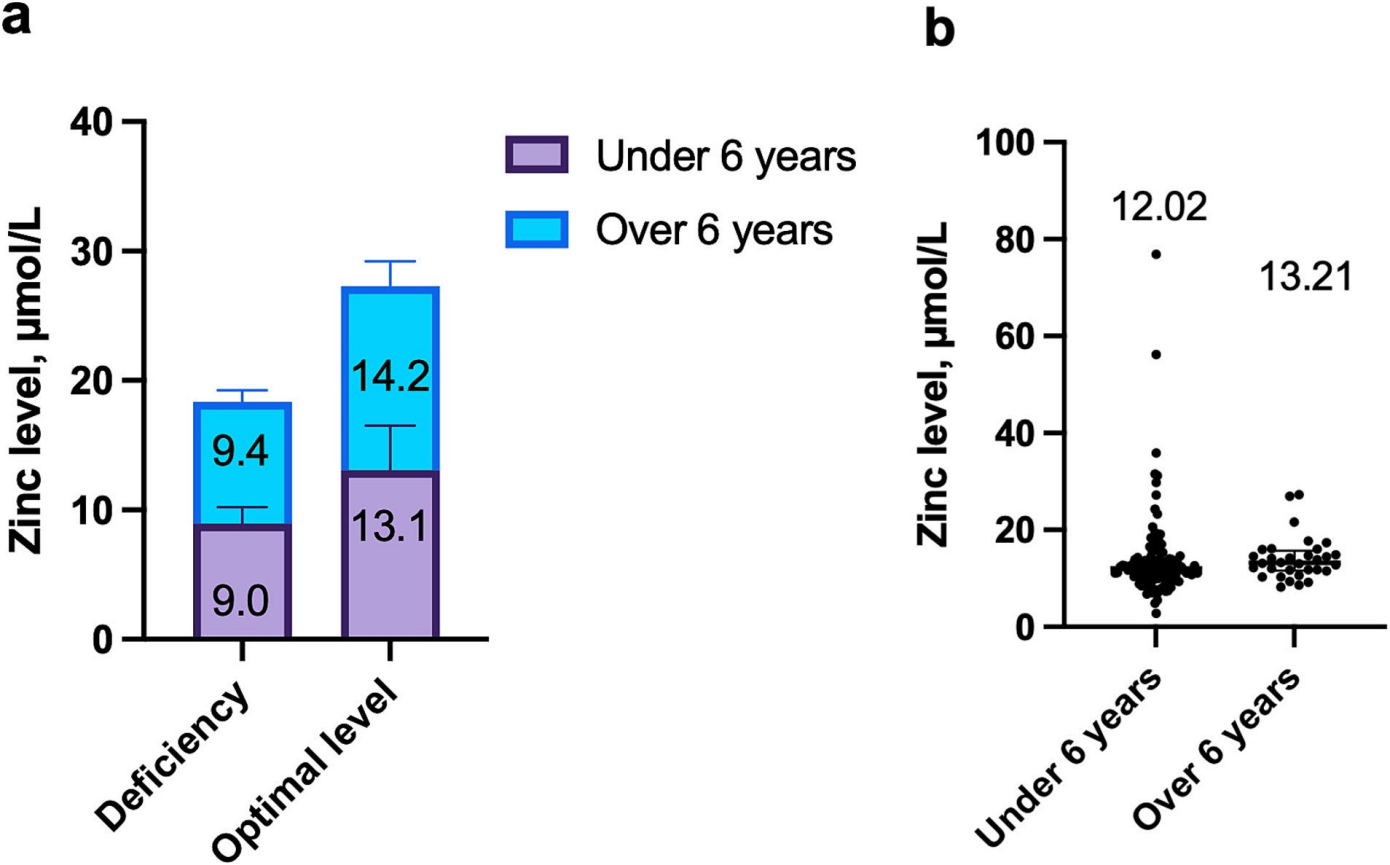
Dependence of zinc levels on age in children with COVID-19: **(a)** Comparison of median zinc concentration in deficiency and optimal levels in children under 6 years and over 6 years, *p* = 0.9576; **(b)** Comparison of zinc levels between children under 6 years and over 6 years, *p* = 0.0983.

Zinc deficiency was identified in 40 children (28.6%), while optimal zinc levels were found in 100 children (71.4%) with COVID-19. The number of children with zinc deficiency did not depend on the age of the patients (Figure 1a). In patients older than 6 years, optimal zinc levels were more commonly observed, though the difference was not statistically significant (78.8% vs. 69.2%, *p* = 0.3790). Conversely, 30.8% of children under 6 years demonstrated zinc deficiency.

Zinc levels were independent of the patients’ age. The median zinc concentration in children under 6 years was 12.0 μmol/L (IQR: 10.4; 14.3), while in children older than 6 years, it was 13.2 μmol/L (IQR: 11.6; 15.7), *p* = 0.0983 (Figure 1b).

### Comparison of clinical characteristics of COVID-19 based on zinc levels

The comparison of clinical characteristics of COVID-19 patients based on zinc levels is presented in Table 1.

**Figure 2:**
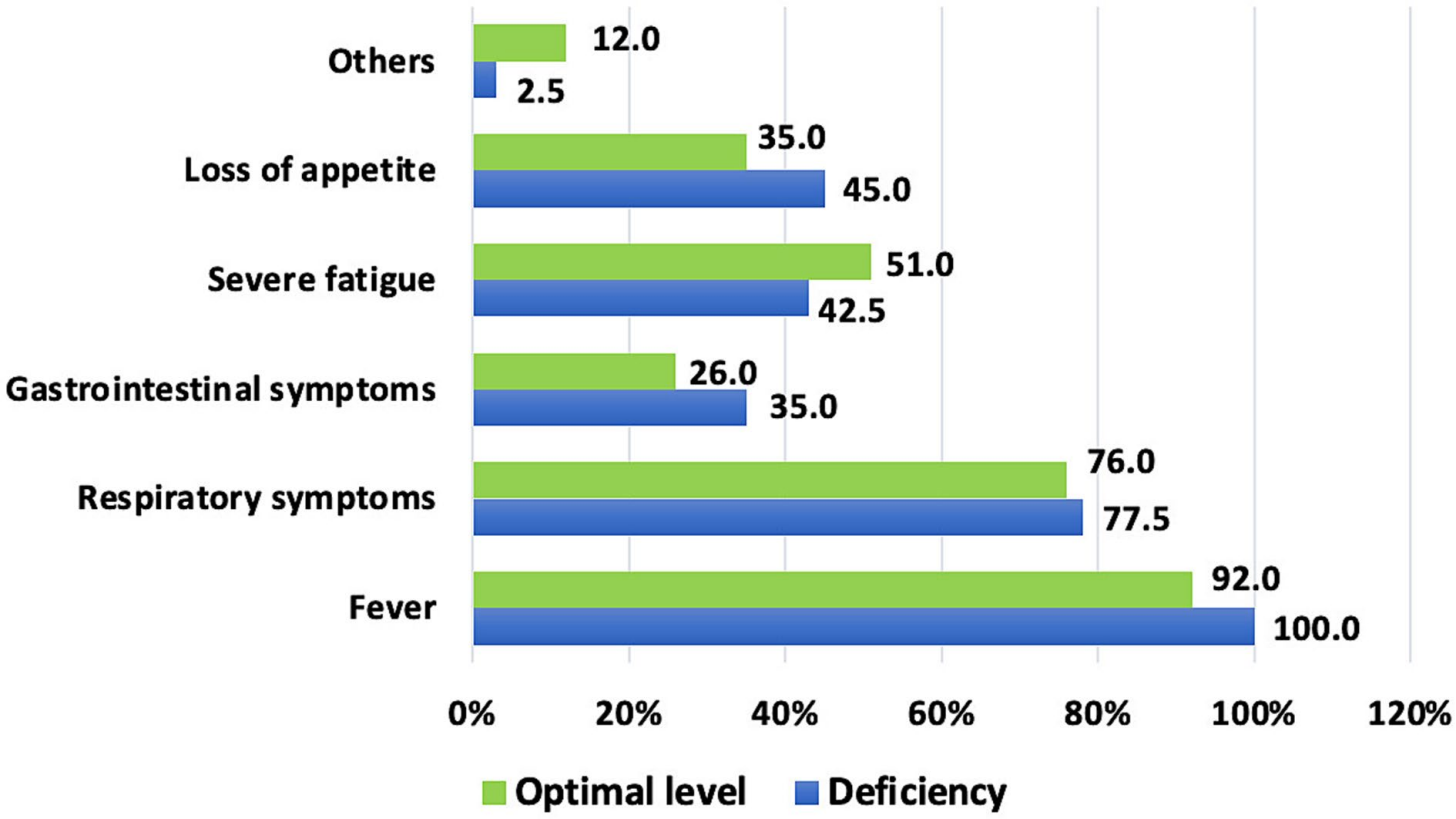
Symptoms of acute SARS-CoV-2 infection depending on zinc status.

Among the symptoms of acute SARS-CoV-2 infection, fever was present in 100.0% of children with zinc deficiency, while it was observed in 92.0% of those with normal zinc levels (*p* = 0.0654). The next most frequent symptoms were respiratory signs, followed by fatigue and loss of appetite, although the frequency of these symptoms did not depend on zinc status (*p* = 0.8502, *p* = 0.3633, and *p* = 0.2704, respectively) (Figure 2).

Comorbidities were observed in both groups with similar frequency (42.5% vs. 54.0%, *p* = 0.2189). Malnutrition and diseases of the nervous and urinary systems were more frequently found in children with low serum zinc levels, but these differences were not statistically significant (*p* = 0.1966, *p* = 0.5329, and *p* = 0.5646, respectively).

The severity of COVID-19 and the duration of hospitalization were not dependent on zinc status.

Levels of leukocytes, neutrophils, lymphocytes were not significantly different between the two cohorts of patients. However, the NLR was found to be higher in children with zinc deficiency than in those with normal levels (*p* = 0.0010). The percentage of patients with a ratio greater than 4 was four times higher in the group with reduced zinc levels, and the difference was statistically significant (*p* = 0.0060).

The median CRP level was significantly higher in children with hypozincemia (*p* = 0.0053). Elevated CRP levels were also more frequently observed in the group with reduced zinc levels.

The median platelet count and the frequency of thrombocytopenia did not differ statistically. A tendency toward a higher frequency of thrombocytosis was observed in patients with low zinc levels (*p* = 0.0692). The mean values of prothrombin time, activated partial thromboplastin time (aPTT), and D-dimer were not dependent on serum zinc status. However, a trend toward more frequent increases in aPTT and D-dimer was observed in children with hypozincemia (*p* = 0.0674 and *p* = 0.0923, respectively). The median fibrinogen level was higher and hyperfibrinogenemia was significantly more common in children with zinc deficiency (*p* = 0.0057, *p* = 0.0338, respectively), although the percentage of its reduced levels was higher in children with optimal zinc values (*p* = 0.0290).

Analysis of creatinine and total protein levels did not show any significant differences based on zinc status.

## Discussion

In our study, the frequency of hypozincemia among children with acute SARS-CoV-2 infection was 28.6%. However, findings from other studies on the prevalence of zinc deficiency in children with COVID-19 have been variable, likely due to differences in study design, patient populations, and methods of zinc level assessment.

Doğan et al. (10) found that zinc deficiency occurred in 23.9% of outpatient children diagnosed with COVID-19, which aligns with our findings, although our cohort consisted of hospitalized patients. In contrast, Ekemen Keleş et al. (33) reported a lower prevalence of hypozincemia (11.0%) among children visiting outpatient clinics due to COVID-19, with a significantly higher average zinc concentration compared to our cohort. On the other hand, another study (37) identified zinc deficiency in 57.4% of children with COVID-19.

Several factors beyond SARS-CoV-2 infection can influence zinc levels. These include older age, male sex, and certain comorbidities, such as diabetes, overweight, and obesity (38–41). In our study population, zinc status did not significantly depend on either the age or sex of the patients. Interestingly, we did not observe zinc deficiency in children with overweight, while 11% of those with an optimal zinc level had excessive weight (*p* = 0.0289). Some studies report a negative correlation between body mass index and zinc levels (42–44), while others have observed a positive impact of zinc supplementation on weight reduction (45, 46). However, other researchers dispute this hypothesis, indicating no significant relationship between zinc status and obesity (47, 48). We did not study zinc intake from food sources or the impact of other factors on zinc status, which could have also influenced the results of our study.

We did not find any difference in the frequency of hypozincemia depending on the severity of COVID-19 or the duration of hospitalization. The conclusions on this issue are contradictory. Ekemen Keleş et al. (33) reported that patients with low serum zinc levels did not have longer hospital stays compared to those with normal zinc levels, which is consistent with our results. In contrast, Yasui et al. (49) demonstrated a connection between zinc deficiency and the severity of COVID-19 in adult patients. Fujita et al. (50) showed that individuals requiring oxygen therapy during the acute phase of SARS-CoV-2 infection had a higher prevalence of zinc deficiency, and the presence of hypozincemia at the time of COVID-19 diagnosis was an independent risk factor for severe disease.

Some studies demonstrate the impact of zinc deficiency on mortality from COVID-19, especially among the adult population (51, 52). Razeghi Jahromi et al. (51) found that patients with zinc deficiency had higher rates of hospitalization, acute respiratory distress syndrome, and mortality. Maares et al. (52) revealed that the overall serum zinc level was significantly lower in patients with COVID-19 compared to the control group, with the lowest levels observed in those who died from the coronavirus disease.

Laboratory analysis showed that the levels of lymphocytes and neutrophils were not dependent on zinc status. In contrast, the NLR was significantly higher in patients with reduced serum zinc levels (*p* = 0.0010). Similar results were observed in the study by Ekemen Keleş (33), which also found no statistically significant differences between zinc levels and the number of leukocytes, neutrophils, or lymphocytes in the blood. Another study showed that the median lymphocyte count was lower in patients with hypozincemia, although the difference was not statistically significant (30). Research involving older adults found that, compared to normal zinc levels, hypozincemia was associated with biomarkers of severe COVID-19, including a higher NLR and lymphopenia (*p* < 0.001) (53).

The association between zinc deficiency and a higher NLR may be explained by zinc’s crucial role in immune system regulation (22). Zinc is essential for lymphocyte proliferation, differentiation, and function, particularly in maintaining T-cell homeostasis and adaptive immunity (24). A deficiency in zinc leads to lymphocyte dysfunction and increased apoptosis, contributing to lymphopenia. At the same time, zinc plays an anti-inflammatory role by regulating neutrophil activity and reducing excessive inflammatory responses. In a state of zinc deficiency, neutrophil activation becomes dysregulated, leading to an increased neutrophil count and a higher NLR, which is considered a marker of systemic inflammation and disease severity in infections such as COVID-19 (9, 54–56). This imbalance between neutrophils and lymphocytes in patients with hypozincemia may contribute to the exacerbation of inflammatory responses and worse clinical outcomes. The limited antiviral response in COVID-19 in the presence of reduced zinc levels may enhance neutrophil infiltration, leading to severe inflammation (57).

The study of inflammation markers during acute viral infection with SARS-CoV-2 is of significant importance, as they can serve as prognostic factors for severe disease progression, mortality, or the development of long-term consequences. In our study, the median CRP was significantly higher in children with COVID-19 and zinc deficiency (*p* = 0.0053). Elevated CRP levels were observed in 68.4% of patients with hypozincemia, and the difference was statistically significant (*p* = 0.0120). Vogel-González et al. (30) also noted that the median CRP was more than twice as high in adult patients with zinc deficiency, and this difference was statistically significant. Razeghi Jahromi et al. (51) also showed a statistically significant negative correlation between zinc levels and CRP using Spearman’s correlation analysis. Almasaud et al. (58) demonstrated a significant negative correlation between serum zinc and inflammation markers, such as leukocytes, CRP, procalcitonin, lactate dehydrogenase, and ferritin. Another study also showed that high CRP levels were significantly associated with hypozincemia, as well as the median ferritin, which was significantly higher in adults with decreased serum zinc levels (53).

Zinc plays an important role in modulating the inflammatory response. Zinc functions as an anti-inflammatory and immunoregulatory micronutrient, influencing various signaling pathways involved in immune activation. It inhibits the NF-κB signaling pathway, a key regulator of pro-inflammatory cytokine production, including IL-6, a major inducer of CRP synthesis in the liver (59, 60). When zinc levels are insufficient, this inhibitory effect is weakened, leading to an increased release of pro-inflammatory cytokines and subsequently higher CRP levels (61).

Additionally, zinc is essential for maintaining the integrity of cell membranes and reducing oxidative stress. A deficiency in zinc results in increased production of reactive oxygen species (ROS) and promotes systemic inflammation, further contributing to elevated CRP levels (20, 62). This mechanism may explain why children with hypozincemia exhibit a more pronounced inflammatory response during SARS-CoV-2 infection.

It is known that zinc acts as an effector of coagulation, anticoagulation, and fibrinolysis, and has properties that regulate hemostasis and thrombosis (63). When analyzing all coagulation markers, we found that the fibrinogen level was significantly higher in patients with zinc deficiency (*p* = 0.0057), and there was also a trend toward thrombocytosis, increased aPTT, and D-dimer levels. Another of our studies showed the age-related characteristics of coagulation markers in children with COVID-19 (64). Vogel-González et al. (30) noted that the average D-dimer level was significantly higher in patients with decreased serum zinc levels. Another study demonstrated that the median platelet count was significantly lower in children with hypozincemia, although D-dimer was higher in the cohort with optimal zinc levels (33).

When analyzing biochemical indicators, we did not observe a statistically significant difference between creatinine and total protein levels. Jothimani et al. (37) also noted that the median creatinine level was not significantly related to zinc levels, unlike lactate dehydrogenase, which was significantly higher in children with zinc deficiency.

### Strengths and limitations of the study

This study provides valuable information about the role of zinc in children with the SARS-CoV-2 viral infection, an area where data remains limited. Most studies focus on adults, which makes this work a significant contribution to understanding the symptoms and course of COVID-19 in pediatric patients. The study features a prospective cohort design with careful monitoring, allowing for a detailed analysis of clinical and laboratory characteristics of COVID-19 based on zinc status.

However, the study has several limitations. The sample size is relatively small, which may affect the generalizability of the results. The study population was selected from a tertiary pediatric hospital, meaning the patients may differ from those in other pediatric hospitals. Additionally, the study did not include a control group consisting of healthy children who did not have COVID-19. Such a group would have provided comparative data on zinc levels in uninfected populations. Another limitation is that serum zinc concentrations were measured after the children were infected. Inflammation can direct zinc to tissues, which can cause a significant decrease in serum or plasma zinc concentrations, regardless of zinc nutriture (65, 66). This phenomenon is typically reversible after recovery. Since infections are known to decrease serum zinc levels without affecting overall body zinc stores, it is unclear whether the observed lower zinc levels are related to a true deficiency or simply part of the normal acute-phase response. In future studies, it would be important to correct for acute-phase response effects, and including CRP as a covariate in zinc analyses could help address this limitation. Although we did not find a correlation between zinc levels and CRP levels, which could suggest that the observed decrease in serum zinc levels may not be solely due to inflammation or the acute-phase response. In future studies, it would be important to correct for acute-phase response effects, and including CRP as a covariate in zinc analyses could help address this limitation.

## Conclusion

Zinc deficiency was found in 28.6% of children with COVID-19 and did not depend on age. In children with hypozincemia, higher levels of inflammation markers were observed, including the neutrophil-to-lymphocyte ratio and CRP. No effect of zinc status on other clinical symptoms of COVID-19 or disease severity was detected. Further studies into the impact of zinc status on the course of COVID-19 and other viral infections require the determination of serum concentrations of the trace element in children before illness and immediately after recovery to retrospectively assess whether low values on admission were transient and likely caused by acute inflammation or whether zinc deficiency is still present. This may improve our understanding of the pathogenesis, symptoms, course, and outcomes of acute infection and predict the likelihood of developing complications.

## Data Availability

The raw data supporting the conclusions of this article will be made available by the authors, without undue reservation.
